# Brain Tumor and Augmented Reality: New Technologies for the Future

**DOI:** 10.3390/ijerph19106347

**Published:** 2022-05-23

**Authors:** Nicola Montemurro, Sara Condino, Marina Carbone, Nadia Cattari, Renzo D’Amato, Fabrizio Cutolo, Vincenzo Ferrari

**Affiliations:** 1 Department of Neurosurgery, Azienda Ospedaliera Universitaria Pisana (AOUP), University of Pisa, 56100 Pisa, Italy; 2 Department of Information Engineering, University of Pisa, 56100 Pisa, Italy; sara.condino@unipi.it (S.C.); renzo.damato@unipi.it (R.D.); fabrizio.cutolo@endocas.unipi.it (F.C.); vincenzo.ferrari@unipi.it (V.F.); 3 EndoCAS Center for Computer-Assisted Surgery, 56100 Pisa, Italy; marina.carbone@unipi.it (M.C.); nadia.cattari@endocas.org (N.C.); 4 Department of Translational Research, University of Pisa, 56100 Pisa, Italy

In recent years, huge progress has been made in the management of brain tumors, due to the availability of imaging devices, which provide fundamental anatomical and pathological information not only for diagnostic purposes. Every year, new surgical intraoperative and planning tools, such as intraoperative MRI, diffusion tensor imaging (DTI), tractography, and intraoperative fluorescent dyes, improve the extent of surgical resection. Similarly, recent advances in brain tumor imaging have offered unique anatomical as well as pathophysiological information that provides new insights into brain tumors. These insights can guide therapeutic decisions and provide information regarding prognosis. In addition, the use of virtual reality (VR) and augmented reality (AR) visualization interfaces in the field of neuroscience and neurosurgery has opened new horizons and new opportunities.

Brain tumors are among the most fatal cancers and account for high morbidity and mortality. Malignant brain tumor incidence declined by 0.8% annually and overall survival remains low [1]. Only 36% of patients survive > 5 years and neurosurgical resection followed by chemotherapy and radiotherapy is advocated as the main treatment [2,3,4]. An increasing number of studies propose commercial AR Head-Mounted Displays (HMDs) as a navigation tool for neurosurgery, despite the technological and human-factor limitations that still prevent achieving high accuracy levels [2,5,6,7]. The most relevant limitations are the perceptual conflicts between the view of the real world and the virtual image, the small field of view, the sub-optimal ergonomics, and calibration issues that hinder the attainment of a robust virtual-to-real alignment. Despite current technological limitations, HMDs are emerging as the most efficient and promising output medium for supporting complex manual surgical tasks typically performed under direct vision, since they allow the surgeon to maintain a ‘surgeon-centered’ point of view and to leave his/her hands-free to operate on the patient [8].

In recent years, several studies in the literature have proposed the use of general-purpose wearable AR displays for neurosurgery [2,9,10]. AR in surgery has enormous potential to help the surgeon in identifying tumor locations, delineating the planned dissection planes, and reducing the risk of injury to invisible structures [8,11,12]. The use of AR HMDs for surgical resection of intracranial meningiomas has already been proposed in [9,10], which provide effective insights into the untapped potential of AR in neurosurgery. However, most reported studies describe ‘proofs of concept’ trials based on the use of consumer-level Microsoft HoloLens headsets beyond their recommended uses. Today, there is a lack in neurosurgery of a clinically tested HMD, designed to be compliant with medical-device regulation, for guiding high-precision tasks.

Novel telemedicine platforms with remote-pointing capabilities and AR interfaces are increasingly being investigated for their applications in several medical and surgical fields [13,14,15]. The use of new technologies aims to improve the guidance of surgical treatment of supratentorial tumors, to enable the surgeon to have adequate access, to decrease the need for repositioning the patient during the surgical procedures, and to reduce the invasiveness of the surgical approach [16,17,18,19,20]. Recent papers proposed the use of AR for a number of procedures: first, to eliminate reintervention to perform the cranioplasty reconstruction for en-plaque cranial vault meningiomas and for all those lesions affecting the bone that needs to be removed and replaced with a customized bone flap; second, to improve safety by ensuring a surgical resection without the occurrence of new neurological deficits; third, the achievement of gross total resection for high-grade glioma or a better resection for low-grade glioma; and fourth, to maximize the therapeutic ratio of radiotherapy [21,22,23,24,25]. In addition, all these technologies could lead to a reduction of blood loss during surgery, a reduction of postoperative pain, and a shorter hospital stay [26].
